# The frequency of maxillary sinus pathologic findings in cone-beam computed tomography images of patients candidate for dental implant treatment

**DOI:** 10.34172/japid.2021.001

**Published:** 2021-02-28

**Authors:** Ashkan Salari, Seyed Ebrahim Seyed Monir, Farzaneh Ostovarrad, Amir Hossein Samadnia, Fereshteh Naser Alavi

**Affiliations:** ^1^Department of Periodontics, Dental School, Guilan University of Medical Sciences, Rasht, Iran; ^2^Department of Oral Radiology, Dental School, Guilan University of Medical Sciences, Rasht, Iran; ^3^Dentist, Dental School, Guilan University of Medical Sciences, Rasht, Iran; ^4^Department of Operative Dentistry, Dental School, Guilan University of Medical Sciences, Rasht, Iran

**Keywords:** Cone-beam computed tomography, Dental implants, edentulous, Maxillary sinus pathology, Posterior maxilla

## Abstract

**Background:**

Maxillary sinus pathologic conditions increase the risk of complications during sinus augmentation surgeries in the posterior maxilla. The present study aimed to determine the frequencies of maxillary sinus pathologic findings on patients’ cone-beam computed tomography (CBCT) images to receive dental implants.

**Methods:**

In this descriptive/cross-sectional study, 140 CBCT images of patients who were candidates to receive dental implants were evaluated for the presence of maxillary sinus pathologic entities during 6 months, were divided into five categories: mucosal thickening of >5 mm, retention cyst, partial or complete opacification of the sinus, polypoidal mucosal thickening, and healthy patients. Age, gender, and dental status were evaluated in terms of relationship with the sinus pathologic findings. Absolute and relative frequencies were used to describe data. The chi-squared test was used to analyze the variables. Statistical significance was set at *P*<0.05.

**Results:**

The frequency of maxillary sinus pathologic entities on CBCT images was 63.5%. The pathologic conditions in descending frequency were as follows: mucosal thickening (31.4%), retention cyst (17.1%), partial or complete opacification of the sinus (9.3%), and polypoidal mucosal thickening (5.7%). The frequency of pathologic findings in the maxillary sinus was higher in the <46-year age group and subjects with partial edentulism; however, the differences were not significant.

**Conclusion:**

In the present study, the most frequent maxillary sinus pathologic entity was mucosal thickening. There was no relationship between age, sex, and dentition status and maxillary sinus pathologic findings.

## Introduction

Dental implant treatment is the first choice to replace lost teeth in the oral cavity due to its high success rate.^1^ The posterior maxilla is among the most challenging areas for implant treatment planning.^2^ After tooth loss in the posterior maxilla, alveolar bone loss, due to thin trabecular bone, leads to decreased bone width and height. However, due to the adequate width of the ridge in the posterior maxilla, there is usually no concern about the ridge width for implant placement. Since the most crucial anatomic landmark in the posterior maxilla is the maxillary sinus, posterior maxilla edentulism is considered a particular case for implant treatment. The maxillary sinus is the first and largest paranasal sinus to form; it is an air-filled cavity in the body of the maxillary bone and drains into the lower and middle meatuses in the nasal cavity.^3,4^ Knowledge about the anatomy, the most frequent variations, and the health or pathologic conditions of the maxillary sinus might play a crucial role in treatment planning for reconstructing the posterior maxilla.^5^ Radiography is an essential adjunctive diagnostic tool to evaluate the residual bone height and width for placing dental implants in the posterior maxilla and evaluating the maxillary sinus. Panoramic and intraoral radiographs cannot provide detailed data about the area in question due to their two-dimensional nature. The cone-beam computed tomography (CBCT) technique provides invaluable data about the height and width of bone and the maxillary sinus condition due to its 3D nature.^6^ A sinus lift procedure might be necessary to reconstruct the edentulous posterior maxilla. Such a procedure is predictable in increasing the height of the posterior maxilla; the survival of implants placed in the maxilla augmented with the lateral or crestal sinus lift procedure is similar to those placed in the native posterior maxilla.^7,8^ However, maxillary sinus augmentation procedures are associated with complications that might compromise the bone grafts’ final outcomes and implant placement procedures. The most common intraoperative complication of maxillary sinus augmentation procedures is the Schneiderian membrane perforation.^9,10^ Pathologic entities in the maxillary sinus might increase the risk of membrane perforation during implant surgery in the posterior maxilla.^11,12^

Previous studies have shown that the sinus membrane perforation might decrease the implant survival rate and bone formation after surgery.^13,14^ However, some studies have not shown any relationship between maxillary sinus pathologic entities before sinus lift procedures and sinusitis risk after surgery.^15,16^ Since pathologic entities in the maxillary sinus might affect the outcomes of implant surgical procedures, knowledge about the different kinds of maxillary sinus pathologic conditions might increase the success of implant surgeries in patients needing such treatments. In this context, the CBCT techniques might be useful as one of the best tools to identify maxillary sinus and its pathologic conditions. Some studies have evaluated maxillary sinus abnormalities.^6,16-20^ This rate was 31.9% in a study by Tadinada et al.^6^ In comparison, it was 73% in the study by Elwakeel et al.^19^ Due to variations in the frequency of maxillary sinus abnormalities in different populations, this study aimed to evaluate the frequencies of pathologic findings in maxillary sinuses on the CBCT images of patients to receive implants in a population in northern Iran.

## Methods

The participants were candidates for dental implant therapy in the maxilla. A total of 140 maxillary CBCT images of patients referring to two specialty oral and maxillofacial radiology clinics were evaluated during six months (summer and autumn seasons). The CBCT images of patients in which one or both maxillary sinuses were fully visible and could be evaluated were included. The exclusion criteria consisted of CBCT images of patients <18 years of age, conditions in which trauma had destroyed the maxilla, and cases in which the patients had undergone maxillary sinus lift procedures or implant placement on one side or both sides.^17^

All the CBCT images were taken with a NewTom 3G (NewTom, Verona, Italy) with FOV=9” for the maxilla and the sinus, with kVp=120, and mA=150. The type of the CBCT machine and the exposure conditions were similar in all the radiology centers. One oral and maxillofacial radiologist evaluated the CBCT images to identify maxillary sinus pathologic entities in all the three coronal, axial, and sagittal cross-sections to eliminate interexaminer errors. The pathologic lesions of the maxillary sinus, which were commonly reported in different studies, were evaluated in the present study, including increased mucosal thickness (>5 mm), partial or complete opacity of the sinus, retention cysts, polypoidal mucosal thickening, and healthy patient.^17-19^ Each patient’s age, gender, and maxillary dental status (complete dentition, partial edentulism, and complete edentulism) were recorded.

The data were analyzed with SPSS 21 for Windows. Absolute and relative frequencies, means, and standard deviations were used to describe the data. The chi-square test was used to analyze the variables. Statistical significance was set at *P*<0.05.

## Results

**Figure 1:**
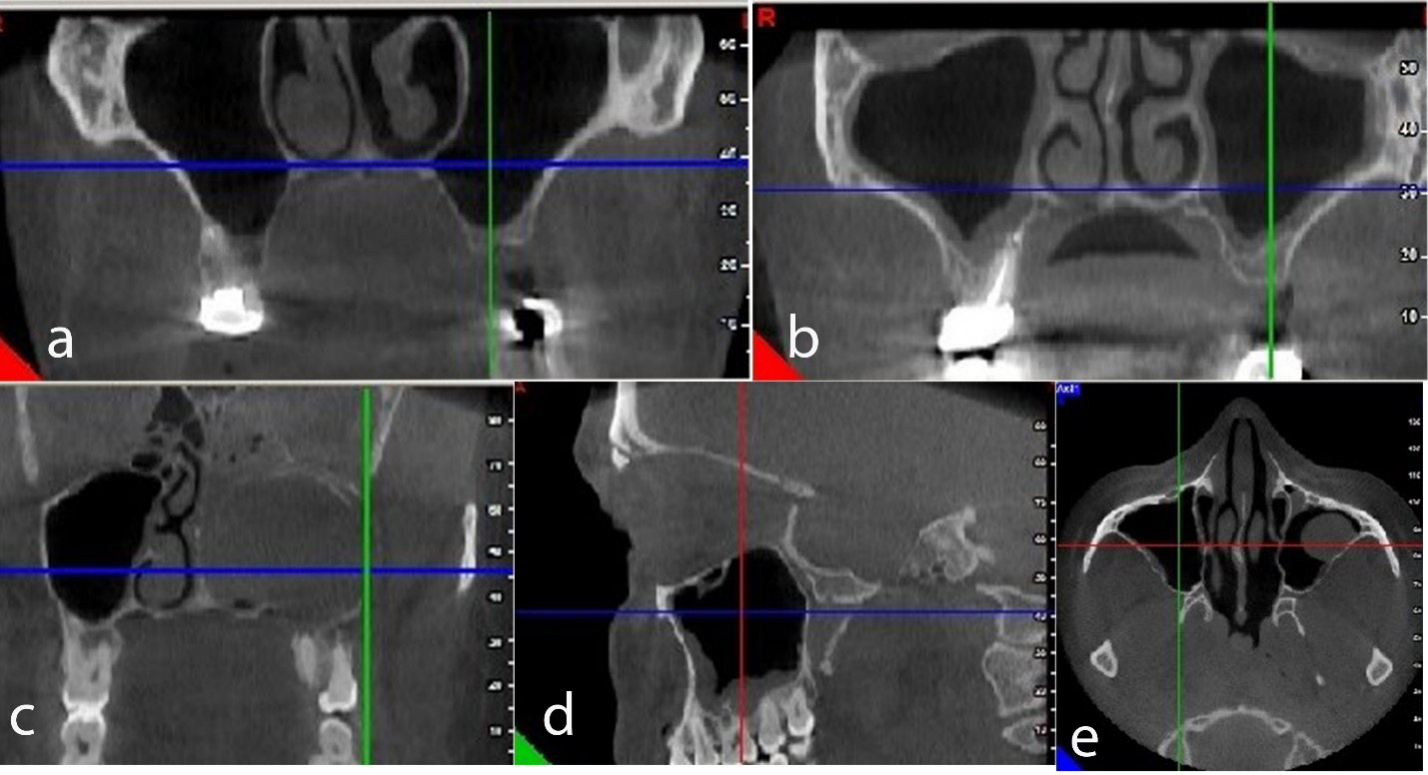
(a) Bilaterally healthy maxillary sinus in axial view. (b) Bilateral mucosal thickening of the maxillary sinus in axial view. (c) Complete opacification of the left maxillary sinus in the axial view. (d) The polypoidal mucosal thickening of the right maxillary sinus in sagittal view. (e) The retention cyst of the left maxillary sinus in axial view.

Of 140 subjects in the present study, 58 (41.8%) were male, and 82 (58.5%) were female, with a mean age of 46 years and an age range of 18–81 years. The patients’ maxillary dentition statuses were as follows: 44 (31.4%) had full dentition, 22 (15.7%) exhibited complete edentulism, and 74 (52.9%) had partial edentulism. No pathologic conditions were identified in 36.5% of CBCT images, while 63.5% showed pathologic findings. Table 1 presents the pathologic findings in maxillary sinuses on the CBCT images of patients. The most and the least frequent pathologic findings in maxillary sinuses were mucosal thickening (31.4%) and polypoidal mucosal thickening, respectively (Figure 1).

**Table 1:** Frequencies of maxillary sinus findings in CBCT images of patients candidate for implant treatment

**Maxillary sinus findings**	**No. (%)**
Mucosal thickening	88 (31.4)
Retention cyst	48 (17.1)
Partial or complete opacification	26 (9.3)
Polypoidal mucosal thickening	16 (5.7)
Healthy	102 (36.5)

The relative frequencies of pathologic findings in maxillary sinuses were higher in patients <46 years of age and in males; however, the difference was not significant. Concerning the dental status, the relative frequency of pathologic findings in maxillary sinuses was higher in patients with partial edentulism than others; however, the difference was not significant (Table 2).

**Table 2:** Frequencies of maxillary sinus pathologic findings based on age, sex, and dentition

**Variables**		**No. (%)**	**No. (%)**	***P*** ** value**
Age	<46	44 (62.8)	26 (37.2)	0.913
	≥46	42 (60)	28 (40)	
Sex	Male	38 (65.5)	20 (34.5)	0.715
	Female	52 (63.5)	30 (36.5)	
Dentition	Full dentition	30 (68.1)	14 (31.9)	0.061
	Partial edentulism	20 (90.9)	2 (9.1)	
	Complete edentulism	48 (64.8)	26 (35.2)	

## Discussion

In the present study, 280 maxillary sinuses on 140 CBCT images were evaluated to assess pathologic entities in these sinuses. The results showed that 36.5% of the CBCT images had no pathologic conditions. In a study by Raghav et al,^17^ 40.3% of the images and in a survey by Manji et al,^18^ 54.9% of the images exhibited no pathologic findings, indicating health.

Previous studies have used different imaging techniques to identify maxillary sinuses’ pathologic conditions, including panoramic, CT, and MRI techniques, with a range of 10.9%–69.1%, mostly between 30% and 60%, consistent with the present study. The MRI technique revealed a higher rate of maxillary sinus abnormality than the CT technique, which might be attributed to the higher diagnostic accuracy of MRI to diagnose soft tissue pathologic entities.^19^ The most frequent pathologic entities in the present study, in descending order, were mucosal thickening, retention cysts, partial or complete opacification of the sinus, and polypoidal mucosal thickening.

The most common maxillary sinus pathologic entity in the present study was mucosal thickening (31.4%), consistent with studies by Elwakeel et al,^19^ Raghav et al,^17^ Manji et al,^18^ and Rege et al.^20^ Only in one study by Tadinada et al,^6^ retention cyst was the most common pathologic finding in maxillary sinuses, different from the present study.

The second most common pathologic entity of maxillary sinus in the present study was the retention cyst (17.1%), which was the second most common pathologic finding in the survey by Rege et al^20^ with 10.1%; however, in the study by Elwakeel et al,^19^ it was the least common pathologic finding with 2.8%. In a survey by Tadinada et al,^6^ it was the most common pathologic finding with 74%. In other similar studies, the retention cyst has not been evaluated as a pathologic finding. The maxillary sinus retention cyst is an expansive inflammatory cyst with pathologic submucosal accumulation of secretions due to the blockage of the secretory ducts of seromucous glands in the sinus mucosa that presents radiographically as a radiopaque dome-shaped structure without a cortical border.^20,21^ One of the possible reasons for differences between different studies is that a retention cyst might be identified at any time of the year; however, these cysts mostly occur in early spring or fall. Their occurrence at this time of the year shows that these cysts are associated with seasonal allergic changes, common colds, moisture, or temperature changes. Therefore, the sample size, use of different imaging techniques, geographic conditions, climate, and the time of the year the study is carried out can be responsible for differences in the study results.

The third most common pathologic finding of maxillary sinuses in the present study was partial or complete opacification of the sinus (9.3%). The frequencies of this pathologic entity in other studies were 13%, 16.6%, 15.4%, and 7.8%, as the third pathologic entity, consistent with the present study.^17-20^

The fourth most common pathologic finding in maxillary sinuses in the present study was polypoidal mucosal thickening (5.7%), consistent with the study by Raghav et al^17^ (7.2%); however, Manji et al^18^ (28.2%) and Elwakeel et al^19^ (16%) reported this pathologic entity as the second most common pathologic finding in their studies. One of the reasons for such a difference is ethnic and geographic differences. According to a survey by Lana et al,^22^ polypoidal lesions are induced by mucous retention cysts and antrochoanal polyps. In the study by Manji et al,^18^ too, retention cyst was not evaluated separately, and retention cysts and nasal polyps were considered polypoidal lesions, which might be one of the possible reasons for a high frequency of this lesion in the present study.

According to the results of the present study, the relative frequency of pathologic findings in the present study was higher in patients <46 years of age than those >46 years of age, with no significant difference; no significant difference was found between age and the pathologic entities of maxillary sinuses. In the study by Rege et al,^20^ no significant relationship was reported between age and maxillary sinus pathologic entities; however, in studies by Raghav et al^17^ and Elwakeel et al,^19^ maxillary sinus pathologic entities were more common in the third and second decades of life, respectively, than other age groups, which might be attributed to racial and geographic differences.

In a present study, similar to the survey by Raghav et al,^17^ there was no significant relationship between gender and maxillary sinus pathologic conditions. However, the relative frequency of maxillary sinus pathologic findings was higher in male patients than female patients, but the difference was not significant.

Another finding of the present study was that the frequency of pathologic findings of the maxillary sinus was higher in subjects with partial edentulism, but the difference was not significant. Sinus mucosal thickening was the most frequent finding in the present study. Such thickening might generally be due to odontogenic irritations and periapical lesions, and periodontal disease. Subjects with residual roots were considered partially edentulous, and the relative frequency of pathologic lesions was higher in partially edentulous patients.

## Conclusion

According to the present study results, the most frequent pathologic finding in the maxillary sinus was mucosal thickening. There was no significant relationship between age, gender, and dental status and maxillary sinus pathologic findings.

## Authors’ contributions

AS and SESM planned the study. FO, AHS, and FN carried out the descriptive cross-sectional study. The statistical analyses and data interpretation were carried out by AS. AS, SESM, FO, AHS, and FN contributed to the literature review. All authors approved the final manuscript.

## Availability of data

The data from the reported study are available upon request from the corresponding author.

## Ethics approval

The protocol of the present study was approved by the Ethics Committee of Guilan University of Medical Sciences under the code IR.GUMS.REC.1396.324. Written informed consent was taken from all the patients for using their scans in this study.

## Competing interests

The authors declare that they have no competing interests regarding authorship and/or publications of this paper.
